# Seasonal and spatial variations in eutrophication and water quality of Lake Aha, Guiyang city, China

**DOI:** 10.1371/journal.pone.0321562

**Published:** 2025-05-07

**Authors:** Yin Su, Bingze Ling, Jintong Ren

**Affiliations:** 1 College of Eco-Environmental Engineering, Guizhou Minzu University, Guiyang, China; 2 Guizhou Key Laboratory of Plateau Wetland Conservation and Restoration, Guizhou University of Engineering Science, Bijie, China; Amity University Amity Institute of Biotechnology, INDIA

## Abstract

This study aimed to analyze the spatial and temporal variations in water quality status and eutrophication level of Lake Aha, influenced by human activities and seasonal changes. Ten indicators were sampled and analyzed at seven sampling points in Lake Aha in different seasons and vertical layers. The eutrophication level and water quality status were evaluated using the Trophic Level Index (TLI) and Water Quality Index (WQI). Results showed that the average TLI and WQI values during the wet season (July) were 31.06 and 92.14, respectively, compared to 28.64 and 109.14 during the dry season (April). Eutrophication was more severe in the wet season than in the dry season, whereas water quality was poorer in the dry season than in the wet season. The main factors driving these patters were temperature and precipitation, respectively. Spatially, the northern part of Lake Aha exhibited higher eutrophication levels than the southern part, while water quality was better in the south than in the north, largely due to the impact of human activities. Significant differences in eutrophication levels and water quality were observed among the seven sampling points, though variations across vertical layers were minimal. Strong positive correlations between key indicators—such as COD_Mn_, TP, and Chl-a—highlighted interdependencies affecting overall water quality, with these factors identified as critical drivers of TLI and WQI. These findings indicate the important impacts of seasonal changes and human activities on the water quality of Lake Aha and suggest the need for targeted water pollution management strategies.

## Introduction

Lake ecosystems play a crucial role in sustaining both natural environments and human societies, serving as vital sources of water, habitats for biodiversity, and hubs for recreational activities [1–3]. The quality of lake water directly affects the socioeconomic well-being of local communities, influencing agriculture, industry, and public health [4–6]. With increasing urbanization, lake ecosystems face growing pressure, necessitating comprehensive assessments to safeguard their ecological integrity and support the communities that rely on them [7]. Effective management of these ecosystems requires a detailed understanding of spatial and temporal variations in water quality parameters [8–10]. Such evaluations are critical for identifying pollution sources, monitoring nutrient dynamics, and implementing targeted interventions to mitigate adverse effects on aquatic life and human health [11,12].

Current methodologies for assessing lake water quality incorporate a range of physical, chemical, and biological indicators [13,14]. Techniques such as remote sensing [15–17], in-situ monitoring [18–21], and ecological modeling [22–25] are widely used to measure parameters like nutrient concentrations, dissolved oxygen levels, and microbial communities. For instance, GIS enable spatial analysis and mapping of water quality parameters [26–28], while the Analytic Hierarchy Process (AHP) structures complex decision-making by evaluating alternatives based on multiple criteria. Fuzzy set analysis integrates qualitative and quantitative data to assess water quality accounting for uncertainty and variability [29–31]. The single-factor assessment, focusing on individual parameters such as pH, dissolved oxygen, and nutrient levels, remains the most common approach [32]. In contrast, index-based methods such as the Water Quality Index (WQI), combine multiple parameters to provide a holistic evaluation of water quality [33]. Recent advancements include artificial neural networks (ANNs), which use machine learning to model complex relationships among water quality variables [34–37]. Despite their utility, these methods have limitations. First, limited spatial and temporal resolution may hinder the detection of subtle water quality variations across seasons or specific locations. Second, reliance on traditional parameters like pH and nutrients may overlook emerging pollutants or ecological indicators. Third, index-based methods oversimplify complex interactions among parameters, potentially masking subtle environmental changes. Finally, challenges in interpreting data uncertainty and variability can undermine assessment reliability.

This study addresses these gaps by conducting a comprehensive evaluation of eutrophication levels and water quality status in Lake Aha, located in the Karst Plateau, a region characterized by karst hilly topography. As a typical karst sub-basin in central Guiyang, Lake Aha is a critical drinking water source heavily influenced by human activities. The Lake is fed by several rivers including the Youyu, Baiyan, Jinzhong, Lanni, and Caichong Rivers. The Youyu River basin features forest and shrubland in its northeastern, northwestern, and southeastern regions, with minimal human impact. However, its northern and southwestern areas are dominated by agriculture--dry fields and rice paddies--where chemical fertilization and tillage signifificantly degrade water quality. Industrial activity, primarily from coal mines, accounts for 99% of the industrial pollution in the Youyu River, with wastewater from numerous small, abandoned mines causing iron and manganese deposition in the riverbed. The Baiyan River basin maintains a relatively healthy ecological environment with limited human influence, though surrounding residential areas, dry fields, and sloped farmland contribute pollution from domestic wastewater and agricultural runoff, particularly in central and eastern regions. The Jinzhong River, flowing through an urban area with numerous industrial enterprises, contributes approximately 70% of the industrial wastewater entering Lake Aha, exacerbating pollution. Its basin, predominantly residential, is heavily impacted by urban wastewater. the upper reaches of the Lanni and Caichong Rivers host herbal planting bases, tree seedling companies, and fishing gardens, alongside residential areas, all of which generate pollution from domestic and agricultural activities. Based on these pollution characteristics, 10 physical and chemical indicators were selected to assess the water quality and eutrophication status of Lake Aha. Physical parameters --pH, dissolved oxygen, turbidity, and transparency-- provide direct insight into the immediate water conditions, while chemical parameters--TOC, chlorophyll-a, permanganate index, ammonia nitrogen, total phosphorus, and total nitrogen--reflect organic pollutants, nutrient levels, and biological activity. The selection of these parameters is consistent with widely accepted water quality guidelines, such as those from the World Health Organization (WHO) and the U.S. Environmental Protection Agency (EPA), and have been used in numerous studies for comprehensive water quality assessment [38,39]. Their selection ensures a holistic evaluation, capturing both immediate physical conditions and underlying chemical composition, which together indicate the lake’s pollution levels. The objectives of the study are: (1) to analyze spatial and temporal variations in water quality parameters across Lake Aha; (2) to evaluate eutrophication status and overall water quality using the Trophic Level Index (TLI) and Water Quality Index (WQI); and (3) to identify the main factors affecting the overall water quality status and nutrient levels. This assessment enhances scientific understanding of eutrophication and water quality in Lake Aha, offering insights for sustainable water resource management in urbanizing regions. By integrating field sampling, laboratory analysis, and statistical modeling, the study provides practical recommendations for improving lake health and evaluating the effectiveness of current management practices.

## Materials and methods

### Study area

Lake Aha, also known as Aha Reservoir (106°37′03″—106°40′39″E, 26°30′38″—26°33′52″N), is located in the southwestern region of Guiyang City, China. This highland artificial lake is representative of its kind. The climate of Lake Aha region is classified as subtropical humid monsoon, with an annual average temperature of 15.3°C. The lake has a total catchment area of 190 km², a water surface area of 4.5 km², an average depth of 13 m, and a maximum depth of approximately 25 m [40]. Five primary rivers feed into the lake--Baiyan River, Jinzhong River, Youyu River, Caichong River, and Lanni River-- with the first three contributing approximately 92% of the catchment area. Based on the lake’s climatic and hydrological characteristics, the dry season spans January to April, the wet season extends from May to September, and the normal water season occurs from October to December [41]. The catchment area is dominated by karst hills, featuring well- developed karst topography. Composed primarily of carbonate rocks, such as Triassic dolomite and limestone, this region exemplifies a typical karst small catchment. In January 2015, Aha Lake Wetland Park was designated a key demonstration site for national wetland park construction. By March of that year, it was rated a national 4A-level tourist attraction and included in the inaugural list of “China Forest Oxygen Bars”.

### Sampling and analysis

**Fig 1:**
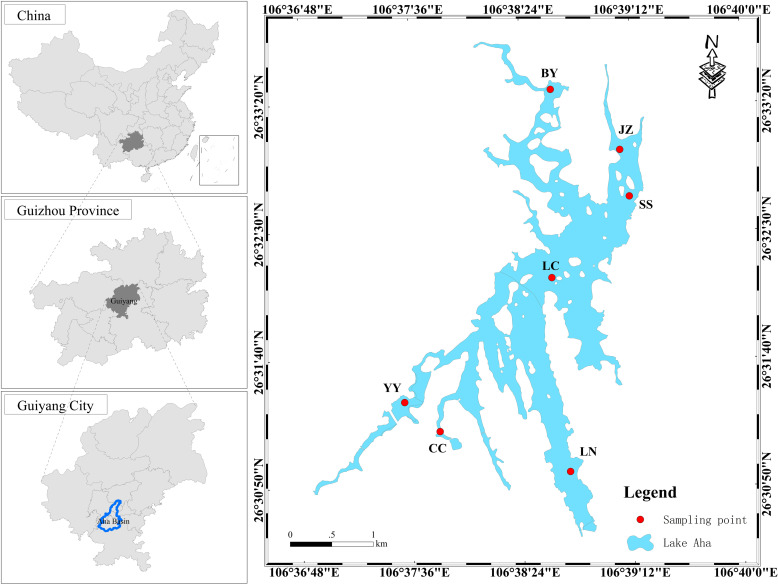
Sampling points and location of Aha Lake. Note: Based on the standard map production of the National Natural Geographic Information Public Service Platform Tiantu website (GS (2024)0650), the base map boundaries have not been modified. (https://cloudcenter.tianditu.gov.cn/administrativeDivision=GS(2024)0650).

In this study, seven sampling points were established across various locations in Aha Lake, including the estuaries of its tributaries, the lake center, southern suburban area of the reservoir. Sampling occurred in April (dry season), July (wet season), and October (normal season) of 2023 (Fig 1). The specific sites were the estuaries of the Baiyan River (BY), Jinzhong River (JZ), Youyu River (YY), Caichong River (CC), and Lanni River (LN), along with the lake center (LC) and southern suburb (SS). At each site, samples were collected from three vertical layers: upper, middle, and lower (Table 1). Samples from the lake’s edge were obtained directly using a sampler, while those from the lake center were collected via a sampling boat. river water samples were stored in glass bottles and refrigerated at 4°C. on-site water samples were acidified to pH < 2 and analyzed on the same day, with the pH adjusted to approximately 7 prior to testing. Standards and parallel samples were included regularly during analysis, achieving a test accuracy of better than ±5%.

**Table 1:** Basic information of Aha Lake sampling sites.

Sampling plots	Sampling depths	Latitude (N)	Longitude (E)	Altitude/m
Baiyan River (BY)	Upper layer (0.5m)	26°33′31″	106°39′15″	1114
	Middle layer(3m)			
	Lower layer (4.5m)			
Jinzhong River (JZ)	Upper layer (0.5m)	26°33′08″	106°39′45″	1105
	Middle layer (5m)			
	Lower layer (8m)			
The southern suburb (SS)	Upper layer (0.5m)	26°32′49″	106°39′48″	1109
	Middle layer (5m)			
	Lower layer (10m)			
The lake center (LC)	Upper layer (0.5m)	26°32′15″	106°39′14″	1106
	Middle layer (5m)			
	Lower layer (8m)			
Youyu River (YY)	Upper layer (0.5m)	26°31′31″	106°38′08″	1111
	Middle layer (5m)			
	Lower layer (8m)			
Caichong River (CC)	Upper layer (0.5m)	26°31′18″	106°39′24″	1110
	Middle layer (3m)			
	Lower layer (4.5m)			
Lanni River (LN)	Upper layer (0.5m)	26°30′48″	106°39′25″	1108
	Middle layer (2m)			
	Lower layer (4m)			

Field measurements of total dissolved solids (TDS), salinity, electrical conductivity (EC), dissolved oxygen (DO), and pH were performed using a YSI ProPlus multiparameter water quality instrument. Turbidity was measured in situ with the HACH 2100P portable turbidity meter, and transparency was assessed using a standard Secchi disk. After collection, lake water samples were digested with NaOH and K_2_S_2_O_8_, and total nitrogen (TN) and total phosphorus (TP) were quantified by colorimetric methods. Following filtration through a 0.45 μm nitrocellulose membrane, ammonia nitrogen and chlorophyll-a were measured. For total organic carbon (TOC) analysis, samples were acidified and aerated to convert inorganic carbon to carbon dioxide, which was then removed, before injection into a high-temperature combustion tube. The permanganate index was determined by adding a known amount of potassium permanganate and sulfuric acid to the sample; after reaction, excess sodium oxalate was used to reduce residual potassium permanganate, and the excess was back-titrated with a standard potassium permanganate solution.

The determination of TN serves as an example of the analytical procedures employed. Detailed methods are provided in references [42–44]. Specific measurement data for each parameter are presented in S1 Table.

(1) Calibration curve preparation. Measure 0.00, 0.20, 0.50, 1.00, 3.00, and 7.00 mL of potassium nitrate standard solution into separate 25 mL stoppered glass colorimetric tubes, corresponding to total nitrogen (as N) contents of 0.00, 2.00, 5.00, 10.0, 30.0, and 70.0 μg, respectively. Then add 5.00 mL of alkaline potassium persulfate solution. Secure the stopper with gauze and string to prevent it from popping out. Place the colorimetric tube in a high-pressure steam sterilizer, heat until the top pressure valve releases air, close the valve, and continue heating until 120°C. Start timing and maintain the temperature between 120–124°C for 30 minutes. Allow it to cool naturally, open the valve to release pressure, remove the outer cover, and take out the colorimetric tube, cooling it to room temperature. Invert the tube and mix the liquid 2–3 times while holding the stopper. Add 1.0 mL of hydrochloric acid solution to the colorimetric tube, dilute with water to the 25 mL mark, and mix well with the stopper. Use a 10 mm quartz cuvette in a UV spectrophotometer, with water as a reference, to measure absorbance at wavelengths of 220 nm and 275 nm.
(2) Sample measurement. Measure 10.00 mL of the sample into a 25 mL stoppered glass colorimetric tube following the same steps as for the calibration curve.
(3) Blank test. With 10.00 mL of water instead of the water sample and follow the same preprocessing and measurement steps as for the sample.
(4) Result calculation. Calculate the mass concentration of total nitrogen in the sample using the calibration curve formula.

### Water quality index (WQI) calculations

The WQI is a statistical tool that integrates individual pollutant indices to provide an overall assessment of a water body’s pollution level. This method evaluates the extent of pollution, identifies key pollutants, and offers a comprehensive measure of water quality. Typically, the WQI assumes that each parameter contributes approximately equally to water quality. The calculation involves averaging the individual pollutant indices, reflecting the combined influence of multiple water quality parameters relative to their respective standards. Higher WQI values indicate greater pollution and poorer water quality, while lower values signify reduced pollution and better water quality. The specific classification standards are detailed in Table 2 [45].

**Table 2:** Water Quality Index Evaluation Standards.

WQI	Grade	Classification Criteria
WQI < 60	Excellent	Most indicators are undetected, and a few detected indicators are within standards
60 ≤ WQI < 80	Good	Most indicators are detected within standards
80 ≤ WQI < 100	Moderate	A few indicators exceed the standards
100 ≤ WQI < 200	Poor	A significant portion of indicators exceed the standards
WQI ≥ 200	Very Poor	A significant portion of indicators exceed the standards by several times or tens of times

In this study, six evaluation parameters were selected: pH, dissolved oxygen, permanganate index, ammonia nitrogen, total phosphorus, and total nitrogen, using Class III water standards from the Surface Water Quality Standard of China [46] as the reference framework [43,45]. Due to the absence of standards for turbidity, transparency, chlorophyll-a, and total organic carbon in GB3838–2002, alternative standards were adopted. For turbidity, a lake standard of 5 NTU was used [47], sourced from the Reuse of urban recycling water-Water quality standard for scenic environment use [48]. For total organic carbon, a standard of 5 mg/L was applied, derived from the Standards for drinking water quality was used [48,49]. For transparency and chlorophyll-a, Class III standard values of 2.5 m and 0.04 mg/L, respectively, were adopted from the “Suggested Control Indicators for Transparency and Chlorophyll in Key Lakes and Reservoirs of Yunnan Province” [50]. The detailed calculation process of WQI is shown in S1 File.

The calculation formula for the single-factor pollutant index is as follows [51]:


$$WQI_{i}=c_{i}/c_{s}$$ (1)

where *WQI*_*i*_ is the single-factor pollutant index for water quality indicator *i*, *c*_*i*_ is the measured value of water quality indicator *i* (mg/L), and *c*_*s*_ is the corresponding environmental standard value for water quality indicator *i* (mg/L).

For the Dissolved Oxygen (DO) Pollution Index, the calculation formula is [52]:


$$WQI_{DO}=\frac{\left|DO_{f}-DO\right|}{DO_{f}-DO_{s}}\;DO_{f}\geq DO_{s}$$ (2)

$$WQI_{DO}=10-9\frac{DO}{DO_{s}}\;DO<DO_{s}$$ (3)

$$DO_{f}=\frac{468}{316+T}$$ (4)

where *WQI*_*DO*_ is the pollution index for dissolved oxygen, *DO* is the actual measured value of dissolved oxygen in mg/L, *DO*_*s*_ is the water quality standard value for dissolved oxygen in mg/L, *DO*_*f*_ is the saturated concentration of dissolved oxygen in mg/L, and *T* is the water temperature in °C.

For the pH Pollution Index, the calculation formula is [52]:


$$WQI_{pH}=\frac{7-pH}{7-pH_{sd}}\;pH\leq 7$$ (5)

$$\begin{array}{c} WQI_{pH}=\frac{pH-7}{pH_{su}-7}\;pH>7 \end{array}$$ (6)

where *WQI*_*pH*_ is the pollution index for *pH*, *pH* is the actual measured value, *pH*_*sd*_ is the lower limit of the water quality standard for *pH*, and *pH*_*su*_ is the upper limit of the water quality standard for *pH*.

For the Water Quality Index, the calculation formula is [53]:


$$WQI=100\times \frac{1}{m}\sum {\mathrm{\backslash nolimits}}_{i=1}^{m}WQI_{i}$$ (7)

where *WQI* is the comprehensive water quality index and *m* is the number of evaluated indicator items.

### Trophic level index (TLI) calculations

The TLI is a quantitative metric used to assess the trophic status or nutrient levels of lakes, reservoirs, and other water bodies. It provides insight into biological productivity and the ecological health of aquatic ecosystems. The TLI is typically calculated using parameters that reflect nutrient concentrations and biological activity, commonly including chlorophyll-a (Chl-a), total phosphorus (TP), total nitrogen (TN), Secchi disk depth (SDD), and the permanganate index (COD_Mn_). For lake Aha, the eutrophication level was assessed using the TLI method [54]. Chlorophyll-a served as the reference parameter to determine the relative weights of other water quality indicators. The TLI was then derived through a weighted calculation, as outlined in Formula (14). The relevant formulas are provided below, and the classification standards are presented in Table 3 [43]. The detailed calculation process of TLI is shown in S2 File.

**Table 3:** Trophic Level Classification Standards for Lakes.

Trophic Level Index	Trophic State Classification
TLI (∑) < 30	Oligotrophic
30 ≤ TLI (∑) ≤ 50	Mesotrophic
50 < TLI (∑) ≤ 60	Slightly Eutrophic
60 < TLI (∑) ≤ 70	Moderately Eutrophic
TLI (∑) > 70	Severely Eutrophic


TLI(Chl−a)=10(2.5+1.0861lnChl−a) (8)

TLI(TP)=10(9.436+1.624lnTP) (9)

TLI(TN)=10(5.453+1.694lnTN) (10)

TLI(SDD)=10(5.118−1.94lnSDD) (11)

$$\begin{array}{c} TLI(COD_{Mn}\text{)=}10(0.109+2.661\ln COD_{Mn}) \end{array}$$ (12)

$$Wj=r_{ij}^{2}\;\;╱\;\;\sum_{j=1}^{m}r_{ij}^{2}\;$$ (13)

$$TLI(\sum )=\sum_{j=1}^{m}WjTLI(j)$$ (14)

Where *TLI(Σ)* is the Trophic Level Index, *TLI(j)* is the composite index of *j*, *W*_*j*_ is the correlative weight, *r*_*ij*_ is the correlation coefficients between the reference *Chl-a* and each parameter *j*, and *m* is the number of indicators. The *r*_*ij*_ value was obtained from the 26 main lake survey data sets for China [55] (Table 4).

**Table 4:** The correlation between Chl.a and the other parameters of lake in China.

Parameters	Chl-a	TP	TN	SD	COD_Mn_
*r*_*ij*_	1	0.84	0.82	-0.83	0.83
*r*_*ij*_^*2*^	1	0.7056	0.6724	0.6889	0.6889

### Statistical analysis

SPSS 19 was used to identify and exclude outliers and to standardize the data by removing dimensional inconsistencies. A Kolmogorov-Smirnov(K-S) test for normality was performed on the water quality indicator data at a significance level of 0.05. The two-tailed asymptotic probability (P) for all parameters exceeded 0.05, confirming that the data followed a normal distribution. At the same significance level 0.05, a one-way analysis of variance (ANOVA) was performed to assess spatial and temporal differences in the WQI and TLI. Additionally, Pearson correlation analysis and Mantel test analysis were conducted to investigate relationships between the water quality status index, eutrophication level index and water quality parameters using a significance level of 0.05. These analyses were implemented in R 4.0.5 with the “corrplot,” “vegan,” “ggcor,” and “ggplot2” packages.

### Research consent statement

This study was approved by Guiyang Aha Lake National Wetland Park Management Office.

## Results

### Temporal and spatial variation characteristics of various water parameters in Aha Lake

#### Seasonal distribution patterns of water parameters in Aha Lake.

**Fig 2:**
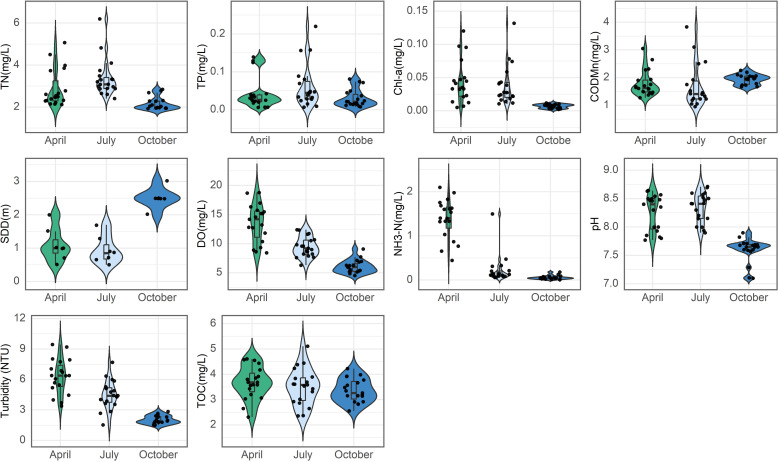
Water indicators summarized at all sampling sites of Aha Lake in different seasons.

TN, TP, Chl-a, permanganate index, and transparency are key parameters of water eutrophication. Fig 2 illustrates the temporal trends of these parameters. TN concentrations showed a bimodal distribution in April, peaking around 2 mg/L and 4 mg/L, with an interquartile range (IQR) of 2.5–4 mg/L. Outliers were present above and below this range. In July, TN distribution became more symmetrical with increased variability (IQR: 2–4.5 mg/L), and outliers extended beyond 6 mg/L. Conversely, October had a narrower distribution (IQR: 1.5–2.5 mg/L), suggesting lower variability and average TN concentrations. For TP, April exhibited a wide, symmetrical distribution centered around 0.05 mg/L, indicating significant variability. In July, TP concentrations were primarily below 0.05 mg/L, but a few extreme values exceeded 0.1 mg/L. October revealed a bimodal distribution with clusters below 0.05 mg/L and around 0.1 mg/L, indicating a complex pattern. In April, Chl-a and COD_Mn_ concentrations averaged around 0.05 mg/L and 2 mg/L. By July, Chl-a showed significant variability, with most values under 0.05 mg/L and some exceeding 0.1 mg/L, while COD_Mn_ primarily ranged from 1.5 mg/L to 2 mg/L, with outliers above 3 mg/L. In October, Chl-a concentrations dropped to below 0.01 mg/L, and COD_Mn_ remained between 1 mg/L and 2 mg/L. Turbidity and transparency in water bodies are inversely related; higher turbidity results in lower transparency. In April, median turbidity was significantly higher at 7.5 NTU (IQR: 6.0–8.5 NTU), with a symmetrical median SDD around 1 meter. By July, median turbidity decreased to 5.0 NTU (IQR: 4.0–6.0 NTU), with a slightly skewed median SDD above 1 meter. In October, turbidity further declined to 3.0 NTU (IQR: 2.5–3.5 NTU), while SDD values became more widely distributed and several outliers were noted, indicating fluctuations in water clarity. In April, the violin plots indicated that DO and NH_3_-N levels were widely distributed, with median concentrations of approximately 14 mg/L and 1.3 mg/L, respectively. DO showed significant outliers above 20 mg/L and below 10 mg/L, while NH_3_-N had outliers exceeding 2 mg/L and below 0.5 mg/L, indicating considerable fluctuations in both parameters. By July, DO values had a narrower distribution, with a median of about 9 mg/L, suggesting reduced variability, although outliers remained present. NH_3_-N similarly showed a median concentration of approximately 0.2 mg/L, reflecting similar trends. In October, DO levels were more evenly distributed, with a median of 8 mg/L and a flatter density plot, indicating significant changes over time. While NH_3_-N was narrowly distributed with a median of 0.05 mg/L and reduced variability, both parameters exhibited outliers. Total Organic Carbon (TOC) reflects organic micro-pollution levels in water. In April, the median TOC concentration is 3.8 mg/L (IQR: 3.5–4.1 mg/L), with whiskers extending from 3.0 to 4.5 mg/L. By July, the median decreases slightly to 3.7 mg/L (IQR: 3.4–4.2 mg/L), while the whiskers range from 2.9 to 4.6 mg/L. In October, the median further drops to 3.6 mg/L (IQR: 3.3–3.9 mg/L), with whiskers from 2.8 to 4.1 mg/L, indicating stable TOC distributions across months. The pH median remains around 8, with minor fluctuations in April and July, while October shows a wider density plot, suggesting increased uniformity and notable outliers down to 7.0.

#### Spatial distribution of various water parameters in Aha Lake.

**Fig 3:**
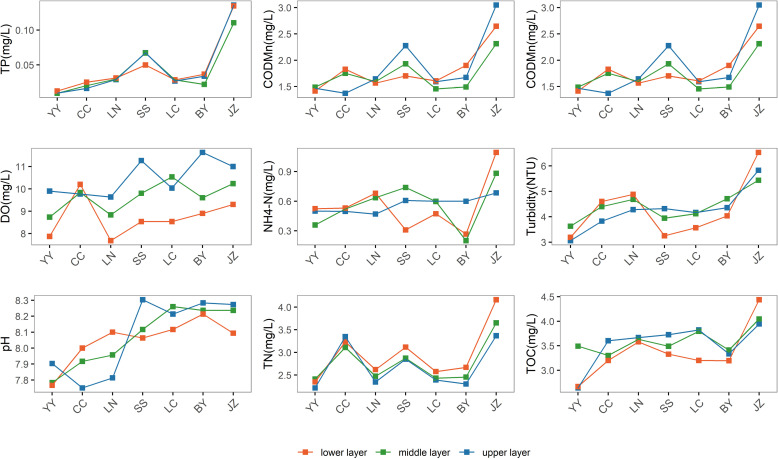
Vertical distribution of water indicators at various sampling points in Lake Aha.

Fig 3 illustrates the spatial distribution of various water parameters. Horizontally, the maximum values for nutrient indicators, including TP, Chl-a, and permanganate index, were observed at the JZ sampling point, followed closely by the SS sampling point, with the lowest values at the YY sampling point. Vertically, the highest TP concentration appeared in the upper layer of JZ, measuring 0.1357 mg/L, while the lowest value was found in the middle layer of YY, measuring 0.0094 mg/L. The difference between the maximum and minimum values is 0.1264 mg/L. At other sampling points, the TP concentration generally followed a pattern of lower layer > middle layer > upper layer. Also, the highest chlorophyll-a concentration appeared in the upper layer of JZ, measuring 0.0855 mg/L. Except for the YY sampling point, where the chlorophyll-a concentrations in the upper, middle, and lower layers were 0.0130 mg/L, 0.0171 mg/L, and 0.0104 mg/L, respectively, the Chl-a concentrations at all other sampling points exhibited a distribution pattern of upper layer > middle layer > lower layer. The maximum permanganate index concentration was found in the upper layer of JZ, at 3.0471 mg/L, while the minimum value was observed in the upper layer of CC, at 1.3728 mg/L. No clear vertical distribution pattern was observed for the permanganate index. The physical indicators at each sampling point show pH values ranging from 7.75 to 8.30, and turbidity ranging from 3.06 NTU to 6.53 NTU. The highest concentrations of TN, TOC, and ammonia nitrogen were all recorded at the JZ sampling point, with the lowest TN and TOC levels observed at the YY sampling point, and the lowest ammonia nitrogen level at the BY sampling point. The maximum DO concentration was found in the upper layer of BY at 11.63 mg/L, while the minimum was recorded in the lower layer of LN at 7.68 mg/L.

### Evaluation of eutrophication levels of Aha Lake based on TLI

**Fig 4:**
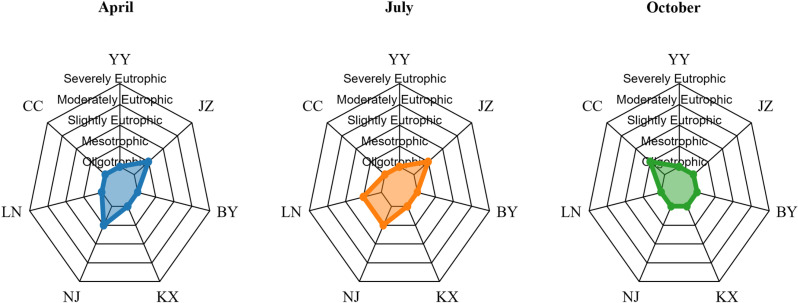
TLI index of different seasons at various sampling points in Lake Aha in 2023.

Fig 4 and Table 5 illustrates the assessment results of waterbody trophic levels across three different months: April, July, and October. These assessments are presented using radar charts, with the axes labeled as YY, JZ, BY, LC, SS, LN, and CC, representing different sampling sites. The trophic levels are categorized from oligotrophic to severely eutrophic. In April, most waterbodies exhibited mesotrophic to slightly eutrophic levels, as indicated by the blue-shaded areas representing the nutrient levels at each sampling site. In July, the majority of the sampling sites displayed distributions ranging from oligotrophic to mesotrophic levels, represented by the orange-shaded areas. In October, the trophic levels of the sampling sites again ranged from oligotrophic to mesotrophic, with the green-shaded areas depicting the conditions during this period. The comparison of data from different months can reveal the impact of seasonal changes on the nutrient status of water bodies. At the same time, the F value in Table 6 is 8.46 and the P value is 0.002569 < 0.05, indicating that season has a significant impact on the TLI index.

**Table 5:** The results of TLI in different seasons.

	YY	CC	LN	SS	LC	BY	JZ
April	16.940	24.740	25.917	32.840	28.160	29.320	42.570
July	20.200	27.750	30.824	35.540	29.430	30.530	43.163
October	10.220	15.850	16.970	20.850	15.160	20.420	22.927

**Table 6:** Variance analysis table of TLI in different seasons.

Source of Variation	SS	df	MS	F	P-value	F crit
Between Groups	734.1882	2	367.0941	8.460428707	0.002568553	3.554557146
Within Groups	781.0116991	18	43.38953884			
Total	1515.199899	20				

**Fig 5:**
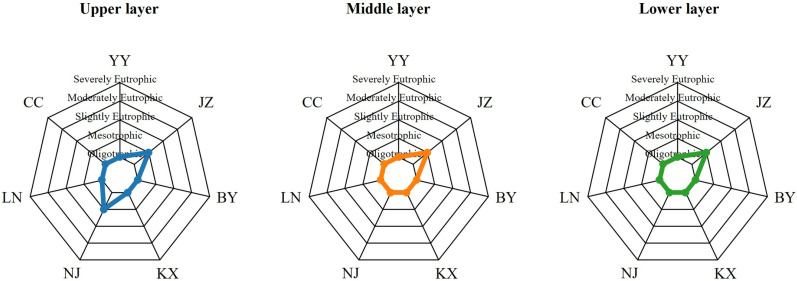
Spatial variation of TLI index at each sampling point in Lake Aha in 2023.

Fig 5 and Table 7 shows the distribution of nutrient levels at different sampling points (YY, JZ, BY, LC, SS, LN, CC) in the upper, middle and lower layers. In the upper layer, most sampling points showed a mesotrophic to slightly eutrophic level, as shown in the blue shaded area representing the nutrient level. In the middle layer, most sampling points showed a distribution from oligotrophic to mesotrophic, as shown in the orange shaded area. In the lower layer, the nutrient levels of the sampling points again ranged from oligotrophic to mesotrophic, and the green shaded area represents the situation during this period. The F value in Table 8 is 0.023 and the P value is 0.977, indicating that there is no statistically significant difference between the groups because the P value is much greater than 0.05. However, the P value in Table 9 is much less than 0.05, so we conclude that there is no significant difference in the TLI index of the vertical layer, but there is a significant difference between the sampling points.

**Table 7:** The results of TLI in different layers.

	YY	CC	LN	SS	LC	BY	JZ
Upper layer	19.013	20.265	25.781	31.418	24.378	23.797	34.685
Middle layer	19.639	22.074	25.741	29.955	24.527	21.337	32.318
Lower layer	18.375	23.210	26.167	27.933	24.139	24.028	32.499

**Table 8:** Variance analysis table of TLI in different layers.

Source of Variation	SS	df	MS	F	P-value	F crit
Between Groups	1.120301	2	0.56015	0.023058	0.977235	3.554557
Within Groups	437.2782	18	24.29323			
Total	438.3985	20				

**Table 9:** Variance analysis table of TLI in different sampling points.

Source of Variation	SS	df	MS	F	P-value	F crit
Between Groups	418.9555	6	69.82591	50.27829	1.12E-08	2.847726
Within Groups	19.44304	14	1.388789			
Total	438.3985	20				

### Evaluation of water quality status of Aha Lake based on WQI

**Fig 6:**
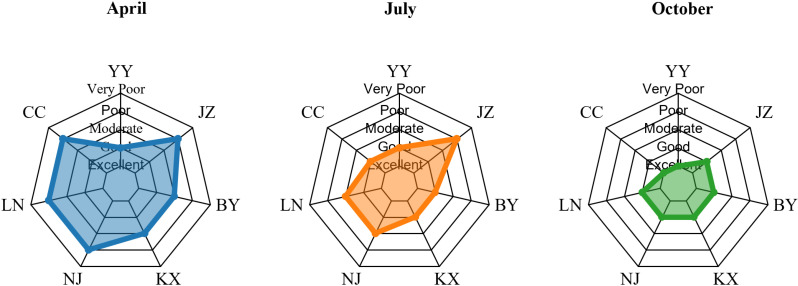
WQI index of different seasons at various sampling points in Lake Aha in 2023.

In Fig 6, the WQI of different sampling points (YY, JZ, BY, LC, SS, LN, CC) during April, July, and October is shown. The WQI values are represented using a radar chart and are divided into five qualities status: excellent, good, moderate, poor, and very poor. In April, the WQI values (in Table 10) of most sampling points ranged from excellent to good, as shown by the blue shaded area. In July, the WQI values of most sampling points were distributed between excellent and moderate, as shown by the orange shaded area. In October, the WQI values ranged from excellent to moderate again, and the green shaded area represents the situation during this period. From Table 11 we can see that the F value of 6.69 and a P-value of 0.00674 indicate statistically significant differences between the groups, as the P-value is less than 0.05. Thus, we reject the null hypothesis, concluding that season significantly affects the water quality status.

**Table 10:** The results of WQI in different seasons.

	YY	CC	LN	SS	LC	BY	JZ
April	69	108	105	111	101	98	172
July	67	75	97	96	82	78	150
October	56	56	60	66	63	62	73

**Table 11:** Variance analysis table of WQI in different seasons.

Source of Variation	SS	df	MS	F	P-value	F crit
Between Groups	7877.428571	2	3938.714	6.6864	0.006737	3.554557146
Within Groups	10603.14286	18	589.0635			
Total	18480.57143	20				

**Fig 7:**
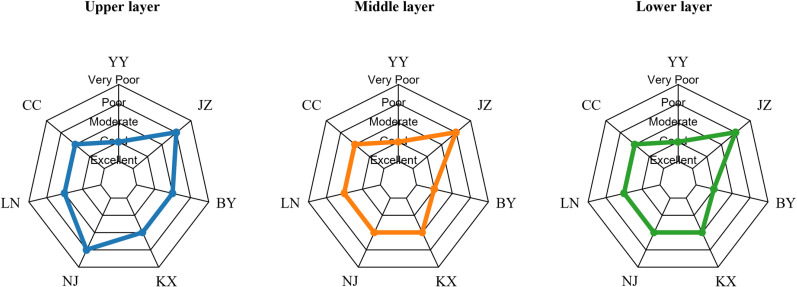
Spatial variation of WQI index at each sampling point in Lake Aha in 2023.

Also, in Fig 7 and Table 12, the WQI of different sampling points (YY, JZ, BY, LC, SS, LN, CC) in different vertical layers (upper, middle, and lower) is shown. In the upper layer, the WQI values of most sampling points ranged from excellent to good, as shown in the blue shaded area. In the middle layer, the WQI values of most sampling points were distributed between excellent and moderate, as represented by the orange shaded area. In the lower layer, the WQI values also ranged from excellent to moderate, and the green shaded area represents the situation during this period. It can be seen that the difference in water quality between the upper, middle and lower layers is not very large. In order to further verify the change of water quality in vertical space, we performed a one-way ANOVA (analysis of variance) analyses of water quality change. As shown in Table 13, the F value is 0.115 and the P value is 0.89188, indicating that at the usual significance level (such as 0.05), there is no statistically significant difference between the groups, that is, there is no significant difference in water quality status in the vertical layers. The P value in Table 14 is much less than 0.05, indicating that the water quality status between the sampling points is significantly different.

**Table 12:** The results of WQI in different layers.

	YY	CC	LN	SS	LC	BY	JZ
Upper layer	65	82	83	107	90	82	136
Middle layer	68	82	87	97	91	69	125
Lower layer	66	83	88	85	81	70	134

**Table 13:** Variance analysis table of WQI in different layers.

Source of Variation	SS	df	MS	F	P-value	F crit
Between Groups	107.8095	2	53.90476	0.115154	0.89188	3.554557
Within Groups	8426	18	468.1111			
Total	8533.81	20				

**Table 14:** Variance analysis table of WQI in different sampling points.

Source of Variation	SS	df	MS	F	P-value	F crit
Between Groups	8037.8095	6	1339.6349	37.812276	7.267E-08	2.847726
Within Groups	496	14	35.428571			
Total	8533.8095	20				

### Relationship between water quality status, eutrophication level and water parameters

**Fig 8:**
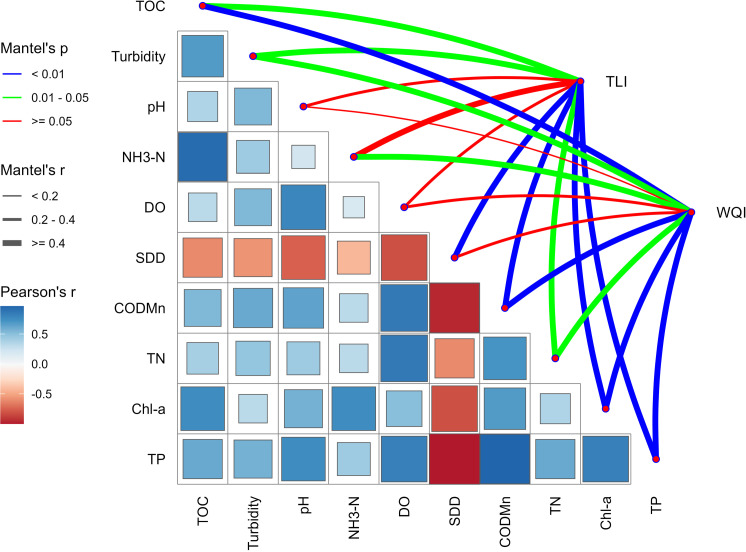
Mantel test of TLI, WQI and water parameters.

The Mantel test can be used to connect the correlation heat map of the water quality physical and chemical parameter matrices on the left side of the Fig 8 with the WQI index and TLI index matrix. This can not only analyze the correlation between water quality status, eutrophication level and water parameters, but also clearly show the correlation between various water quality physical and chemical parameters through the test chart.

The results show that TP, Chl-a, and permanganate index exhibited significant correlations with both the WQI and TLI (P < 0.01), indicating that these three parameters are the main water quality factors influencing WQI and TLI. Based on the thickness of the correlation lines, TOC, TP, Chl-a, and permanganate index had correlation coefficients of r ≥ 0.4 with the WQI, while TP, Chl-a, permanganate index, and SDD had correlation coefficients of r ≥ 0.4 with the TLI. The correlation coefficients between other water quality parameters and the WQI or TLI were r < 0.4. Overall, TP, Chl-a, and the permanganate index are the key parameters impacting the water quality status and eutrophication level of Aha Lake.

Within the Pearson correlation coefficient matrix, several significant relationships among water parameters are evident: TOC shows a strong positive correlation with COD_Mn_, with a correlation coefficient close to 0.7. This suggests that TOC and COD_Mn_ tend to increase or decrease together in the studied samples, possibly reflecting shared sources of organic pollutants or similar environmental behaviors. SDD exhibits a significant positive correlation with Chl-a, with a correlation coefficient near 0.6. The relationship between transparency (SDD) and Chl-a implies that increased transparency may be associated with higher algal biomass, a common occurrence in eutrophic waters. NH_3_-N shows weaker correlations with other variables such as DO and pH, with generally lower correlation coefficients.

## Discussion

### Variation analysis of the WQI and TLI in Lake Aha

Significant spatial (horizontal) and seasonal effects (*P* ＜ 0.05) were observed on the TLI of Lake Aha. Temporally, TLI values across all sampling points followed the pattern July > April > October (Table 5). During the wet season in July, with an average water temperature of 26.7°C, TLI values exceeded 30 at the JZ, SS, LN, and BY sampling points, indicating a mesotrophic state. The remaining points—LC, CC, and YY--were classified as oligotrophic. In the dry season in April, with an average water temperature of 20.1°C, only the JZ and SS points reached mesotrophic levels, while the others remained oligotrophic. In the normal season of October, with an average temperature of 17.0°C, all sampling points had TLI values below 30, indicating oligotrophic conditions. The decline in water temperature from 26.7°C in the wet season to 17.0°C in the normal water season corresponded to a reduction in the level of eutrophication, consistent with Herb and Stefan [56], who identified temperature as a key driver of seasonal eutrophication dynamics in water bodies. Spatially, the TLI rankings across the seven sampling points were JZ > SS > BY > LC > LN > CC > YY, indicating that eutrophication was most severe at JZ and least pronounced at YY. This spatial variation primarily reflects anthropogenic pollution impacts. The JZ River, which flows through the urban area of Guiyang, is heavily influenced by organic-rich domestic wastewater discharges, resulting in significantly higher eutrophication levels compared to other rivers [57]. The SS point, located near JZ, exhibited the second-highest eutrophication level, while the CC and YY points, less impacted by human activities, showed lower pollution levels. There was no significant vertical variation in TLI levels across the sampling points in Aha Lake.

The WQI index of the seven sampling points in Lake Aha also exhibited significant seasonal and horizontal spatial differences (*P* ＜ 0.05), but no notable vertical variation. From the perspective of seasonal variability, WQI values followed the pattern April > July > October (Table 10). In April (dry season), WQI values exceeded 100 at all points except YY and BY-- where water quality status was good to moderate-- indicating poor water quality elsewhere. In July (wet season), only the JZ point had a WQI above 100, indicating poor water quality status, while other points ranged below 100, reflecting good to moderate conditions. In October (normal season), all WQI values were below 80, denoting good to excellent water quality status. precipitation levels in Aha Lake were lowest in April (23.6 mm), highest in July (138.5 mm), and moderate in October (51.2 mm). Moderate precipitation can dilute pollutants and reduce the lake’s pollution load [58], whereas insufficient rainfall, as in April, reduces water volume, shrinks littoral wetlands, and weakens pollutant dilution [59]. Conversely, excessive rainfall, as in July, can increase soil erosion in the watershed, transporting non-point source pollutants and nutrients from soil sediments into the lake via runoff, thereby elevating pollution levels [60,61]. From the perspective of horizontal spatial variability, WQI rankings were JZ > SS > LC > LN > CC > BY > YY, indicating that JZ was the most polluted and YY the least polluted among the sampling points.

### Factors influencing the water quality status and eutrophication level of Aha Lake

In natural aquatic environments, water quality parameters do not operate independently; instead, they interact as a composite factor. This study used the Mantel test to comprehensively evalute correlations between various water quality parameters and the WQI and TLI matrices. The results revealed that the permanganate index (COD_Mn_), TP, and Chl-a concentration exerted the most significant influence on both WQI and TLI. This finding aligns with the suggestion by Pesce and Wunderlin [51] to calculate WQI using 3–5 key water quality parameters, including COD_Mn_ and TP. The spatiotemporal variation in the water quality status and eutrophication levels of Aha Lake is driven by two primary factors. Seasonally, differences in water quality status and eutrophication are largely governed by meteorological conditions: precipitation predominantly affects water quality status, while temperature primarily influences eutrophication levels. These two indices exhibit opposing trends--higher eutrophication but better water quality in the wet season, and lower eutrophication but poorer water quality in the dry season—consistent with Wang [43], who reported a significant negative correlation between WQI and TLI. On the other hand, the spatial differences in water quality status and eutrophication levels are mainly attributed to human activities. Among the sampling points, the JZ exhibited the worst water quality status and eutrophication levels, while the YY showed the best conditions. JZ is impacted by both agricultural runoff and urban pollution, as it flows through densely populated areas where domestic sewage is discharged, exacerbated by an urban drainage system that has not fully separated rainwater from sewage. In contrast, the YY riverbanks are predominantly lined with forests, shrubs, or wasteland, characterized by dense vegetation and high coverage. Girmay et al. [62] found that vegetation cover exceeding 65% significantly reduces nitrogen and phosphorus losses in surface runoff, a key factor explaining the pronounced spatial variation in water quality status and eutrophication levels across Lake Aha.

## Conclusions

Influenced by human-induced pollution, the spatial distribution of eutrophication levels in Lake Aha exhibits a clear trend: levels are highest in the northern rivers, lower in the lake center, and lowest in the southern rivers. Water quality status follows a parallel pattern, with the poorest conditions in the northern rivers, intermediate conditions in the lake center, and the best conditions in the southern rivers. Specifically, the northern JZ River shows the most severe eutrophication and poorest water quality, while the southern YY River displays the lowest eutrophication and best water quality. Seasonally, water quality status, affected by precipitation and temperature, ranks from worst to best as follows: April (dry season)> July (wet season)> October (normal season). In contrast, eutrophication levels from highest to lowest, are: July (wet season)> April (dry season)> October (normal season). Among the 10 water quality parameters in Aha Lake, TOC is strongly correlated with the COD_Mn_, and SDD is strongly correlated with Chl-a. The Mantel test indicates that TP, Chl-a, and COD_Mn_ exhibit the strongest relationships with the TLI and WQI, identifying them as the key factors influencing water quality status and eutrophication levels in Aha Lake. Overall, the water quality characteristics of Aha Lake are shaped by both seasonal variations and human pollution activities, highlighting the need for targeted and region-specific water pollution management strategies.

## Supporting information

S1 Table The measured values of each parameter. (XLSX)

S1 File Detailed description of the calculation process of WQI. (ZIP)

S2 File Detailed description of the calculation process of TLI. (ZIP)

S3 File Supporting Information. (ZIP)
